# Which papillary thyroid microcarcinoma should be treated as “true cancer” and which as “precancer”?

**DOI:** 10.1186/s12957-019-1638-0

**Published:** 2019-05-31

**Authors:** Krzysztof Kaliszewski, Dorota Diakowska, Beata Wojtczak, Zdzisław Forkasiewicz, Dominika Pupka, Łukasz Nowak, Jerzy Rudnicki

**Affiliations:** 1 Department of General, Minimally Invasive and Endocrine Surgery, Wroclaw Medical University, Borowska Street 213, 50-556 Wroclaw, Poland; 2 Department of Nervous System Diseases, Faculty of Health Science, Wroclaw Medical University, Wroclaw, Poland; 3 Department of Surgery Didactics, Wroclaw Medical University, Wroclaw, Poland

**Keywords:** Papillary thyroid microcarcinoma, Aggressiveness, Metastasis

## Abstract

**Background:**

Papillary thyroid microcarcinoma (PTMC) generally is a cancer with excellent prognosis, but the term “cancer” sounds severe and harsh, which can elicit emotional and physical responses from patients. To eliminate the word “cancer,” the term noninvasive follicular thyroid neoplasm with papillary-like nuclear features (NIFTP) was introduced. However, not all PTMCs can be classified as NIFTP. Sometimes, very aggressive PTMC cases might be observed. Some authors suggest that one of the risk factors for poor prognosis is lymph node metastasis. The aim of the study was to evaluate some clinicopathological features of PTMC as the risk factors for lymph node metastasis.

**Material and methods:**

We performed a retrospective chart review and selected 177 patients with PTMC. To analyze the cases with potentially aggressive behavior, we enrolled PTMC patients with lymph node metastases (pN1, central, and/or lateral) and evaluated some of their clinicopathological features.

**Results:**

The logistic regression analysis results demonstrated significantly higher rates of multifocal or bilateral tumor occurrence in the PTMC patients with pN1 than in the patients with pN0 (*P* < 0.0001 for both). In addition, the occurrence of thyroid tumors with sizes above 0.5 cm was a significant risk factor for lymph node metastasis (*P* < 0.0001). The results of the ROC analyses showed that the presence of multifocal or bilateral tumors and tumor sizes above 0.5 cm were significant predictors of lymph node metastasis (*P* < 0.0001 for all).

**Conclusions:**

Multifocal and bilateral PTMC tumors with diameters above 0.5 cm should be treated aggressively as “true cancer” and might benefit from lymph node dissection. Unifocal PTMC tumors with diameters equal to or below 0.5 cm may be treated less aggressively.

## Introduction

Papillary thyroid microcarcinoma (PTMC) is a thyroid cancer with small tumors that are 1.0 cm maximum in diameter [1]. The term “PTMC” was introduced for the first time by the World Health Organization (WHO) in 1989 [1]. The majority of PTMCs are clinically silent and sometimes might be found from histopathological examinations after a thyroidectomy performed due to benign thyroid diseases [2]. Additionally, PTMCs are found in autopsies of individuals who died of non-thyroid-related diseases. PTMC is also the most common type of malignant thyroid tumor with an increasing prevalence, but also with an excellent prognosis. Therefore, the term “carcinoma” is thought by many authors to be too severe and harsh since it can elicit emotional and physical responses from the patients [3, 4]. On the other hand, some very aggressive PTMC cases might be observed [5]. Choi et al. stated that no definite biological or clinical parameters currently exist to distinguish low-risk indolent PTMC from potentially aggressive PTMC [6]. According to some authors, one of the risk factors for aggressive PTMCs is lymph node metastasis [7–18]. These cases may benefit mostly from lymph node dissection. Sometimes, we diagnose palpable metastatic neck lymph nodes as the first sign of PTMC; these tumors are known as occult papillary thyroid microcarcinomas.

To promote not aggressive surgical approach and save patients’ psychological distress of cancer diagnosis, a new name for indolent PTMC such as noninvasive follicular thyroid neoplasm with papillary-like nuclear features (NIFTP) was introduced [19]. However, the term NIFTP in histological diagnosis is strictly stringent [20]. For example, the tumor has to present encapsulation, purely follicular architecture, the presence of nuclear features of papillary thyroid cancer (PTC), and absence of capsular and vascular invasions. They strictly formulated the inclusion and exclusion criteria because not every PTMC can be classified as NIFTP.

It is suggested that the increasing prevalence of PTMCs with excellent prognoses is caused by the increased use of ultrasound examinations of the thyroid. The management of these “clinically silent thyroid incidentalomas” is still controversial [3]. However, the most common subsequent clinical evaluation is ultrasound-guided fine needle aspiration biopsy (UG-FNAB). If the diagnostic result of this procedure is estimated as category V or VI according to The Bethesda System for Reporting Thyroid Cytopathology (TBSRTC) and PTMC is highly suspected, then the first clinical dilemma appears. The main question is to what extent surgery should be performed. Which of the procedures, hemithyroidectomy or thyroidectomy, is the better therapeutic option? The next clinical dilemma regards lymph node dissection. The problem is more straightforward if we have enlarged lymph nodes of the neck (measuring more than 1.0 cm in the short axis diameter), and subsequently, metastases are microscopically confirmed. In such situations, therapeutic lymph node dissection is obviously recommended. A different situation is when the neck lymph nodes are not pathologically enlarged. This clinical status is often highlighted because some authors say that microscopic neck lymph node metastases are present in over 60–80% of PTMC patients [9, 21]. Therefore, the question is if prophylactic node dissection should be taken into consideration.

Regarding the increasing rate of PTMC occurrences with often indolent clinical behavior, various options for disease management have emerged. However, so far, the most common treatment that is recommended by many authors is surgery. Regardless of the PTMC’s “clinically silent behavior,” researchers recommend either thyroidectomy or minimum hemithyroidectomy with isthmectomy. The second management option recommended by other researchers is active surveillance with surgical treatment when tumor progression occurs [22, 23].

After considering all the mentioned dilemmas, a fundamental question appears: which PTMC cases should be treated as “true cancer” and which as “precancer”? To answer this question, we evaluated some clinicopathological features of PTMCs in stage pN1 to select tumors that should be treated aggressively as “true cancers,” and then, we evaluated some clinicopathological features of PTMCs in stage pN0, which may be treated less aggressively as “precancers.”

## Materials and methods

We performed retrospective chart reviews of 4716 patients who were admitted and surgically treated in one center between 2008 and 2017. Among the patients, 434 (9.2%) had thyroid malignancies; finally, we selected 177 (3.75%) patients with papillary thyroid cancers, which were described as the tumors with 1.0 cm maximum in diameter on the basis of histopathological examinations and thereby were classified as papillary thyroid microcarcinomas (PTMCs). All of the patients were staged in accordance with TNM staging criteria (tumor-node-metastasis) proposed by AJCC 8th Edition [24]. Preoperative thyroid ultrasonography, UG-FNAB, and cytological examinations were performed in all cases. After surgery, the final histopathological classification was performed according to World Health Organization Guidelines. The surgical tissue specimens were fixed in 10% buffered formalin and diagnosed histopathologically at the Department of Pathomorphology, Wroclaw Medical University. Representative blocks were selected. Because of the fact that papillary thyroid carcinoma can be multifocal, the adjacent and opposite lobe was sampled and all pale areas were processed. Lesions with a diameter maximum of 1.0 cm or less were processed in their entirety; however, a minimum of 5–8 blocks were taken from each lesion. Serial sectioning and careful cutting of the representative tissue sample was done. A routine method of specimen processing was performed. Sections were cut in 4-μm thickness on which conventional hematoxylin and eosin (H&E) staining sections were prepared. H&E sections were evaluated by two experienced thyroid lesion pathologists to confirm the diagnosis, features of the tumor, and the extent of the malignant process. Patients were diagnosed as PTMC, if the largest tumor diameter was equal or less 1 cm. To analyze the cases with potentially aggressive behavior, we enrolled PTMC patients with lymph node metastases (pN1, central, and/or lateral) and evaluated some of their clinicopathological features. Of the 177 patients with PTMC, 116 (65.5%) were diagnosed with thyroid malignancies before surgery, 63 (35.6%) subjects presented with multifocal tumors, and 15 (8.5%) presented with bilateral tumors (Table 1). The PTMC patients were divided into two groups according to the histopathological diagnosis of their lymph node metastases: the pN0 group (*n* = 115)—without metastasis, and the pN1 group (*n* = 62)—with lymph node metastasis.

**Table 1:** Backgrounds and clinicopathological characteristics of PTMC patients (*n* = 177)

Parameters	*N* (%) or mean ± SD
Gender:	
Male	23 (13.0)
Female	154 (87.0)
Age (years)	48.3 + 14.1
Age	
< 55 years	109 (61.6)
≥ 55 years	68 (38.4)
Diagnosis of malignancy	
Before surgery	116 (65.5)
After surgery	61 (34.5)
Diagnosed as multifocal	
No	114 (64.4)
Yes	63 (35.6)
Diagnosed as bilateral	
No	162 (91.5)
Yes	15 (8.5)
Tumor size	
< 0.5 cm	81 (45.8)
≥ 0.5 cm	96 (54.2)
Lymph node metastasis	
No	115 (65.0)
Yes	62 (35.0)
Type of surgery	
Total	126 (71.2)
No-total	51 (28.8)
Reoperation	
No	131 (74.0)
Yes	46 (26.0)

*PTMC* papillary thyroid microcarcinoma, *SD* standard deviation

### Statistical analysis

Descriptive data were presented as numbers of observations and percentages or as averages and standard deviations (± SD). Student’s *t* tests for independent samples or chi-square tests were applied for group comparisons. The Kaplan-Meier method and log-rank test were performed to compare the disease-free survival distribution of N0 and N1 patients. Multivariable logistic regression analysis was used to confirm the selected factors that were associated with an increased risk of lymph node metastases. The diagnostic potential of each independent variable was determined by receiver operating characteristic (ROC) analysis and was expressed in terms of the area under the ROC curve (AUC). The accuracy, sensitivity, specificity, positive predictive value (PPV), negative predictive value (NPV), likelihood ratio of positive results (LR(+)), likelihood ratio of negative results (LR(−)), and Youden Index were also calculated. All calculated *P* values were two-sided, and *P* < 0.05 was considered significant. Statistical analyses were performed using Statistica 13.1 (TIBCO Software Inc., CA, USA).

## Results

To date, none of the 177 patients in our series who underwent surgery and subsequent observation died of thyroid cancer. By questionnaire, we confirmed that 5 patients died of not PTMC-related disease.

**Fig. 1:**
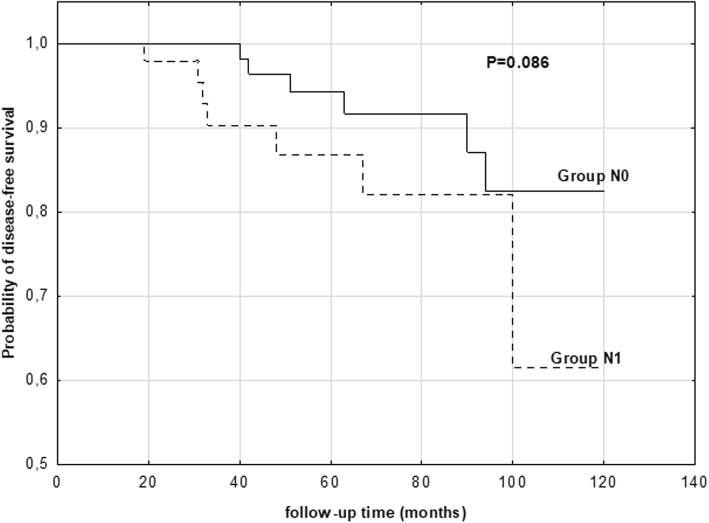
Relationship between lymph node metastasis and the probability of disease-free survival of PTMC patients

We created the model of lymph nodes status as a predictor of disease-free survival. The probabilities of disease-free survival in the PTMC patients with N0 or N1 lymph node metastasis at the term of observation are showed on Fig. 1. The proportion of disease-free survival was highest in N0 patients (83%) than in N1 patients (62%). These differences were not significant; however, the trend was observed (83% vs. 62%), *P* = 0.086.

Multivariable logistic regression analysis was performed for the selection of clinical and pathological predictors of lymph node metastasis (Table 2). We demonstrated significantly higher rates of multifocal or bilateral tumor occurrence in the PTMC patients in the pN1 group than in the patients in the pN0 group (*P* < 0.0001 for both) (Table 2). In addition, the occurrence of thyroid tumors above 0.5 cm in size was a significant risk factor for lymph node metastasis (*P* < 0.0001), whereas age, gender, and capsular invasion were not found to be significant predictors of lymph node metastasis (*P* > 0.05).

**Table 2:** Multiple logistic regression analysis of clinical and pathological factors that can be predictors of lymph node metastasis (pN0/pN1, 0/1) in the patients with PTMC. Results were also confirmed by chi-square test

Variables	Patients with pN0 (*n* = 115)	Patients with pN1 (*n* = 62)	*P* value (*χ*^2^ test)	Logistic regression analysis		
				OR	± 95% CI	*P* value (Wald test)
	*N* (%)	*N* (%)				
Gender			0.065	0.93	0.42–2.06	0.858
Male	11 (9.6)	12 (19.3)				
Female	104 (90.4)	50 (80.7)				
Age			0.303	0.96	0.51–1.80	0.890
< 55 years	74 (64.4)	35 (56.5)				
≥ 55 years	41 (35.6)	27 (43.5)				
Diagnosed as multifocal			< 0.0001*	9.40	4.6–19.3	< 0.0001*
No	94 (81.7)	20 (32.3)				
Yes	21 (18.3)	42 (67.7)				
Diagnosed as bilateral			< 0.0001*	2.90	0.0–12.8	< 0.0001*
No	115 (100.0)	47 (75.8)				
Yes	0 (0.0)	15 (24.2)				
Tumor size			< 0.0001*	65.80	15.1–287.3	< 0.0001*
< 0.5 cm	79 (68.7)	2 (3.2)				
≥ 0.5 cm	36 (31.3)	60 (96.8)				
Capsular invasion			0.047	2.54	0.98–6.56	0.052
No	106 (92.2)	51 (82.3)				
Yes	9 (7.8)	11 (17.7)				

*PTMC* papillary thyroid microcarcinoma, *CI* coefficient interval, *statistically significant

**Fig. 2:**
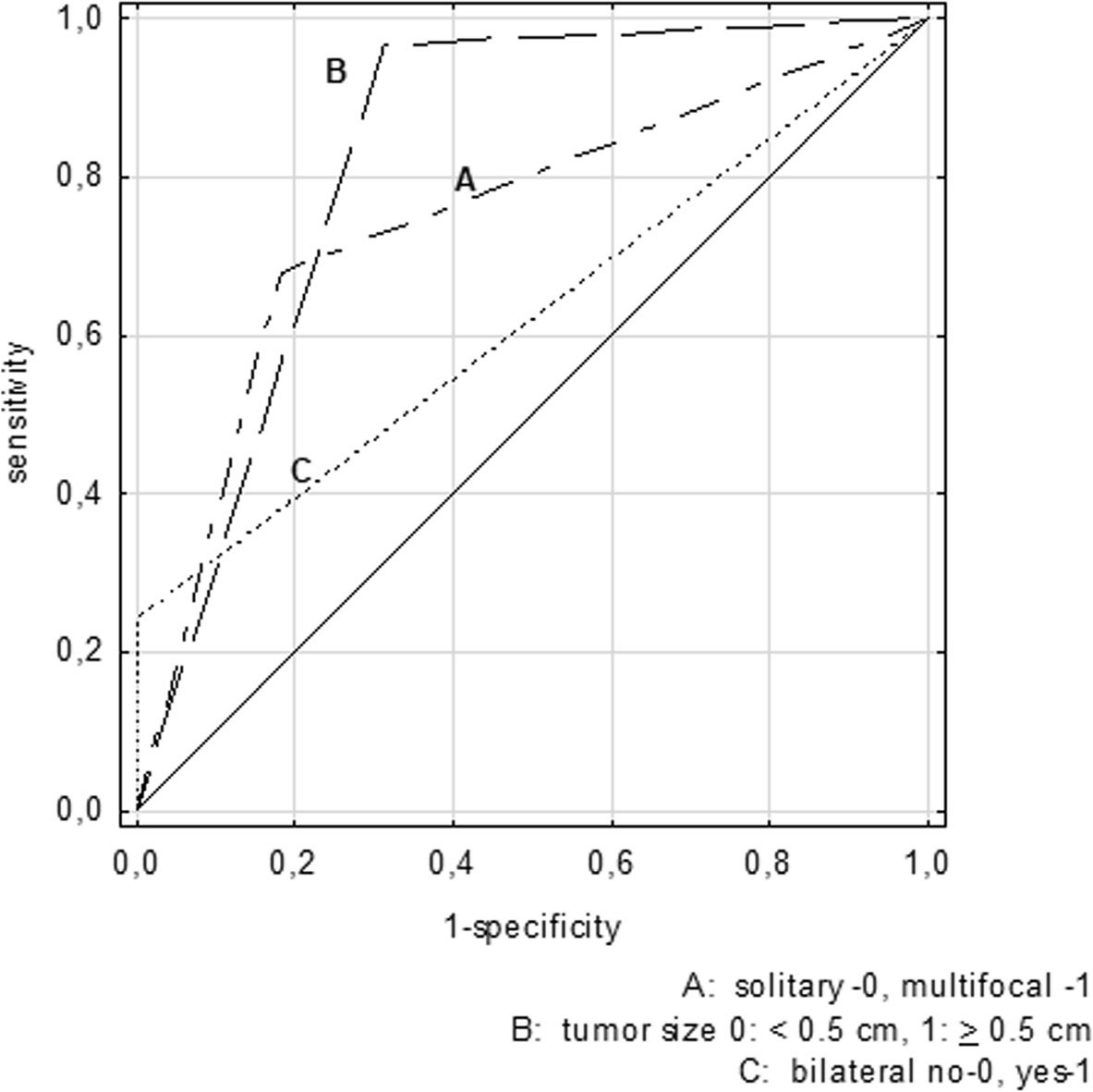
Comparison of receiver operating curves (ROC) for the presence of multifocal or bilateral tumors or tumor size above 0.5 cm. The performance of a marker unable to discriminate between pN0 and pN1 patients, whose area under the ROC curve (AUC) is equal to 0.5 was marked with a constant line

The diagnostic potential of three selected factors was evaluated in terms of their capability to differentiate PTMC patients with lymph node metastasis from PTMC patients with pN0. The results of the ROC analysis showed that multifocal or bilateral tumors and tumor sizes above 0.5 cm were significant predictors of lymph node metastasis (*P* < 0.0001 for all) (Table 3). The presence of tumors above 0.5 cm in size and multifocal tumors were found to be the best predictors of lymph node metastasis in PTMC (Fig. 2).

**Table 3:** Diagnostic potential of multifocal and bilateral tumors presence and tumors size above 5 mm as indicators of lymph node metastasis in PTMC patients

ROC analysis	Presence of multifocal tumors	Presence of bilateral tumors	Presence of tumor ≥ 0.5 cm
AUC (± 95%CI)	0.747 (0.668–0.827)	0.621 (0.529–0.713)	0.827 (0.767–0.888)
*P* value	< 0.0001	< 0.0001	< 0.0001
SE	0.041	0.047	0.031
Sensitivity	0.677	0.242	0.968
Specificity	0.817	1.000	0.687
Accuracy	0.768	0.734	0.785
LR (+)	0.667	1.000	0.625
LR (−)	0.825	0.710	0.975
PPV	0.183	0.000	0.313
NPV	0.323	0.758	0.032
Youden Index	0.495	0.242	0.665

*PTMC* papillary thyroid microcarcinoma

## Discussion

In light of the recent evidence regarding the safety of nonsurgical management of patients with PTMC, the term “precancer” to describe tumors that are often indolent tumor may be introduced. Even the American Thyroid Association (ATA) guidelines allow for an active surveillance management approach instead of immediate surgery in patients with low-risk PTMC [25, 26]. Recently, we have observed that clinicians for some select individuals who are diagnosed with PTMC offer active surveillance instead of surgical treatment [27, 28]. According to the NCCN Guidelines Version 1.2019 rather, minimum lobectomy is recommended; however, some criteria like no prior radiation exposure, no distant metastases, no cervical lymph node metastases, and no extrathyroidal extension must be presented [29].

Currently, the term NIFTP is commonly used and is suggested for many well-differentiated thyroid tumors [19]. The main goals of introducing the term NIFTP were firstly, deleting the word “carcinoma” describing an indolent form of PTMC and secondly, to prevent overdiagnosis and overtreatment [20]. In our study, after accurate reanalysis of all PTMC specimens by two experienced pathologists, who performed histopathological examinations, according to their suggestions, we did not decide to name these tumors NIFTP, because of some pathologic characteristics such as capsular invasions or the presence of true papillae defined as exclusion criteria [19].

With increasing awareness of the overdiagnosis with overtreatment phenomena of PTMCs along with the recent clinical observations and ATA guideline recommendations, it seems very useful to introduce data about PTMCs that may be treated as a “precancer.” This information may help endocrinologists, radiologists, and surgeons to understand the real clinical and pathological nature of PTMC cases. This is why some authors suggest changing the PTMC terminology, which in their opinion, may replace patients’ perceptions of their cancer diagnosis [4]. On the other hand, very aggressive PTMC might be sometimes observed [3]. One of the most unfavorable clinicopathological features of aggressive PTMCs is lymph node metastasis [7, 8]. Jiang et al. suggested that the presence of central or lateral lymph node metastases in papillary thyroid cancer (PTC) cases affects the prognosis and treatment of the individuals [8]. Moreover, these metastases increase the rate of regional recurrence and mortality, especially in elderly patients [30]. Some authors suggest that almost 80% of PTMC patients develop lymph node micrometastases, which are usually diagnosed in postsurgical histopathology [21]. However, lymph node metastases are diagnosed in only 30% of patients with PTC during clinical examinations [21]. It was also presented in Xue’s review that the accuracy of preoperative ultrasonography for the diagnosis of lymph node metastases is not very high and can be as high as 48.3% [26]. It is very important to assess the clinicopathological features that could be significantly specific for lymph node metastasis and that are usually undetected during routine physical examinations. Making an accurate selection of PTMC cases that could be treated as “precancer” requires high sensitivity in examinations to even exclude cases of lymph node micrometastases. These excluded cases should be treated more aggressively as they are considered “true cancers.” Xue et al. revealed that the sensitivity of ultrasound ranged from 22.6 to 55% in predicting central lymph node metastases, which means that almost half of the patients with metastases were incorrectly diagnosed [26].

Generally, in Poland, the diagnoses and treatment experiences widely vary for patients with PTMC. The majority of these tumors are asymptomatic and are often discovered by diagnostic imaging examinations ordered due to issues that are unrelated to thyroid pathology. This suspicion of PTMC starts a diagnostic and treatment cascade, which almost always leads to surgery. These medical procedures often result in physical and emotional side effects in patients diagnosed with PTMC [4]. Considering the rate of overdiagnosis and to avoid stressful and uncomfortable situations, we should inform the patients about the potential consequences of thyroid testing and its possible final diagnosis. However, if we suspect PTMC after thyroid diagnostic tests, we should discuss all clinical aspects of the discovered tumor with the patient. One very useful and informative message is that clinicians often describe PTMC as a “small and slow-growing tumor.” We also should inform the patients that currently, more and more researchers offer patients the option of active surveillance instead of surgery. According to some authors’ results, even lymph node metastases are not a crucial problem [22, 23]. These authors conclude that the rate of occurrence of lymph node metastases in individuals with PTMC who are with active surveillance is comparable to the rate observed in patients with PTMC who underwent thyroidectomies immediately after diagnosis. Moreover, the authors also estimated that the outcomes of thyroid surgery are the same regardless of whether surgical treatment is undertaken immediately after PTMC diagnosis or after the first observation of tumor progression. Oda et al. state that delayed surgery in patients with PTMC, who initially followed active surveillance, is safe, and the results are comparable to individuals who underwent immediate surgical management [27]. Currently, we can observe that some authors recommend active surveillance for PTMC instead of surgical treatment. Next, we should discuss the current ATA guideline recommendations that allow performing a hemithyroidectomy instead of a total thyroid resection in the majority of PTMC cases. However, we observe that most of the patients, even after discussions with accurate and precise clinical information, choose surgical treatment options. Some patients prefer less invasive surgical procedures, such as a hemithyroidectomy, to avoid taking levothyroxine supplements for the rest of their life. However, these individuals should also be informed that all surgical options, even partial thyroid resection, will require thyroid hormone supplements [31, 32].

As of now, some basic fundamental questions still remain: “What we can do to avoid stressful and difficult clinical situations that elicit emotional and physical responses from patients?” and “What should be the proper management of PTMC?” Some authors controversially suggest abandoning fine needle aspiration biopsies of all thyroid nodules equal to or below 1.0 cm in diameter. Such an approach should reduce the number of unnecessary diagnostic procedures for PTMC [29]. Additionally, the authors of this study believe that the main focus of PTMC concerns is overdiagnosis rather than overtreatment. Neck ultrasound examinations are widely and easily accessible, which often cause unsuspected diagnoses of the thyroid nodule. Subsequently, if we have even one unfavorable ultrasound feature to describe a newly diagnosed incidentaloma, almost every clinician will recommend a UG-FNAB [33]. Some authors suggest that involving patients in the shared decision-making process about whether to perform a UG-FNAB might be an effective strategy in reducing the number of unnecessary diagnostic procedures associated with incidental PTMCs [34, 35].

Some authors have suggested that metastases frequently affect both the central and ipsilateral neck lymph nodes, even in patients with PTMC [9]. Other authors added that skip lesions, i.e., the involvement of the lateral compartment without central lymph nodes, are rare, but their incidence is similar to that of larger PTCs [36]. However, other authors say that patients with multifocal PTMC tumors are more likely to have lymph node metastases [9]. Gur et al. analyzed all types of PTC (including PTMC) and estimated that the risk of local recurrence, lymph node metastasis, and distant metastasis are increased in multifocal PTC patients, so they concluded that multifocality is a poor prognostic factor for PTC [37]. In our study, we demonstrated significantly higher rates of multifocal or bilateral tumor occurrence in PTMC patients with lymph node metastases than in patients without metastases (pN1 vs. pN0). Some authors have assessed that the location of the primary PTMC tumor did not predict the pattern of lymph node metastases, which is similar to observations of PTCs [38]. In our study, we did not conduct analyses between tumor locations and lymph node metastasis patterns. Wada et al. showed that the size of the primary PTMC tumor slightly influenced the frequency of lymph node metastasis. The frequency was 55.7% for tumors that were 0.5 cm in diameter and 73.7% for those that were > 0.5 and 1.0 cm in diameter. The authors did not observe an effect on nodal recurrence [9]. In our study, we noticed that tumor sizes above 0.5 cm were a significant risk factor for lymph node metastasis. The presence of tumors above 0.5 cm in size and multifocal lesions were found to be the best predictors of PTMC lymph node metastasis. Because of the results of our study, which showed that multifocal or bilateral tumors and tumor size above 0.5 cm were significant predictors of lymph node metastasis, it may implicate some additional pathologist’s procedures. In some individual cases, i.e., with lymph node metastases and larger tumors (≥ 0.5 cm), the whole resected thyroid tissue should be pathologically examined to exclude multi- and bilaterality of PTMC.

There are several limitations in this study. Firstly, it is limited by its retrospective design. Secondly, the study is also limited by the number of multifocal and bilateral PTMC. However, multifocal and bilateral PTMC is not very often observed. The next limitation of our study is its single center analysis. Thus, multicenter and large cohorts study should be performed to identify the larger number of multifocal and bilateral PTMC.

## Conclusions

Multifocal or bilateral PTMC tumors with diameters above 0.5 cm should be treated aggressively as “true cancer.” Because lymph node metastases are observed more commonly in such cases, thyroidectomy and selected central/lateral lymph node dissection are recommended. These cases might benefit mostly from this procedure. Unifocal PTMC tumors with diameters equal to or below 0.5 cm in some individuals may be treated less aggressively; a hemithyroidectomy with isthmectomy is an acceptable procedure.

Our study helps clinicians to more safely and effectively select patients for whom aggressive treatment is recommended or is not necessary. Additionally, for some select individuals with PTMCs that are stage N0, their diagnoses could be explained as a “precancer” for emotional comfort.

## Data Availability

Not applicable.

## Abbreviations

ATA: American Thyroid Association
PTC: Papillary thyroid cancer
PTMC: Papillary thyroid microcarcinoma
TBSRTC: The Bethesda System for Reporting Thyroid Cytopathology
UG-FNAB: Ultrasound-guided fine needle aspiration biopsy
